# Neurofibroma with adenosis in the mammary gland: a case report

**DOI:** 10.1186/s40792-023-01673-0

**Published:** 2023-05-29

**Authors:** Hirokazu Yamazaki, Kei Koizumi, Mana Watahiki, Daiki Takatsuka, Yuko Asano, Mana Goto, Norihiko Shiiya, Satoshi Baba

**Affiliations:** 1 First Department of Surgery, Hamamatsu University School of Medicine, 1‑20‑1 Handayama, Higashi‑ku, Hamamatsu, Shizuoka 431‑3192 Japan; 2 Department of Pathology, Hamamatsu University School of Medicine, 1‑20‑1 Handayama, Higashi‑ku, Hamamatsu, Shizuoka 431‑3192 Japan

**Keywords:** Benign breast tumor, Neurofibroma, Adenosis, Ultrasound, Mammography

## Abstract

**Background:**

Neurofibroma of the breast is extremely rare, with only a few reported cases. Here, we report a case of solitary neurofibroma of the breast in a 95-year-old woman.

**Case presentation:**

A 95-year-old woman presented with a palpable mass in the left breast. Mammography revealed a well-defined mass. A 1.6-cm round mass was found in the lower outer quadrant of the left breast on ultrasonography. The internal echo of the tumor was a mixture of relatively uniform hypoechoic areas with posterior enhancement and heterogeneous hyperechoic areas. She underwent a core needle biopsy. The pathological findings revealed a spindle cell lesion with no malignant findings. At 2 months follow-up, repeat breast ultrasonography showed that the mass had enlarged to be 2.7 cm in size. A repeat core needle biopsy, however, revealed no particularly new information. Because the tumor was growing and a definite diagnosis was not made, lumpectomy was performed. We found bland-spindled cells with shredded-carrot collagen bundles. Immunohistochemical antibody markers (S100, SOX10, and CD34) were positive for the spindle cells. Some of the tumors maintained the bilayer nature of luminal cells and myoepithelial cells, which might be the reason for internal heterogeneity on ultrasound. A histological diagnosis of neurofibroma with adenosis was made. At 6 months follow-up, no recurrent lesions were found.

**Conclusions:**

Ultrasound and pathological images revealed an extremely rare case of neurofibroma combined with adenosis. Tumor resection was performed because it was difficult to make a definitive diagnosis using needle biopsy. Even when a benign tumor is suspected, short-term follow-up is necessary, and if an enlargement is observed, early tumor resection is recommended.

## Background

Neurofibromas are benign tumors that were first described by Smith in 1849 and later by von Recklinghausen in 1882 [1]. Neurofibromas involving the breast are mostly superficial and located in the dermis [2] with neurofibromatosis type 1 being the most common [3–5]. However, neurofibromas of the breast are extremely rare, with only a few reported cases. We report a case of solitary neurofibroma with adenosis of the breast in a 95-year-old woman that was difficult to diagnose using core needle biopsy.

## Case presentation

**Fig. 1:**
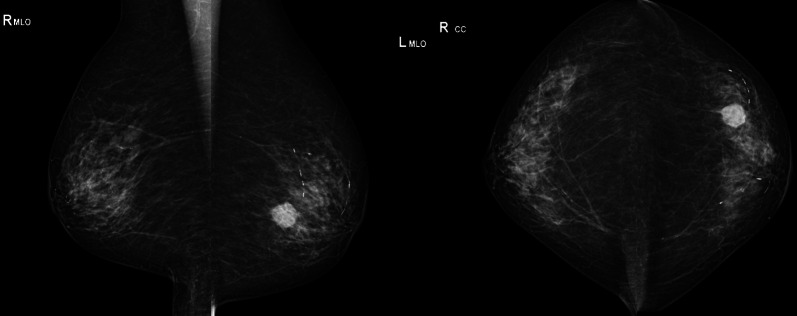
Mammography revealed well-defined masses in the middle and outer areas of the left breast. There was no evidence of tumor calcification

**Fig. 2:**
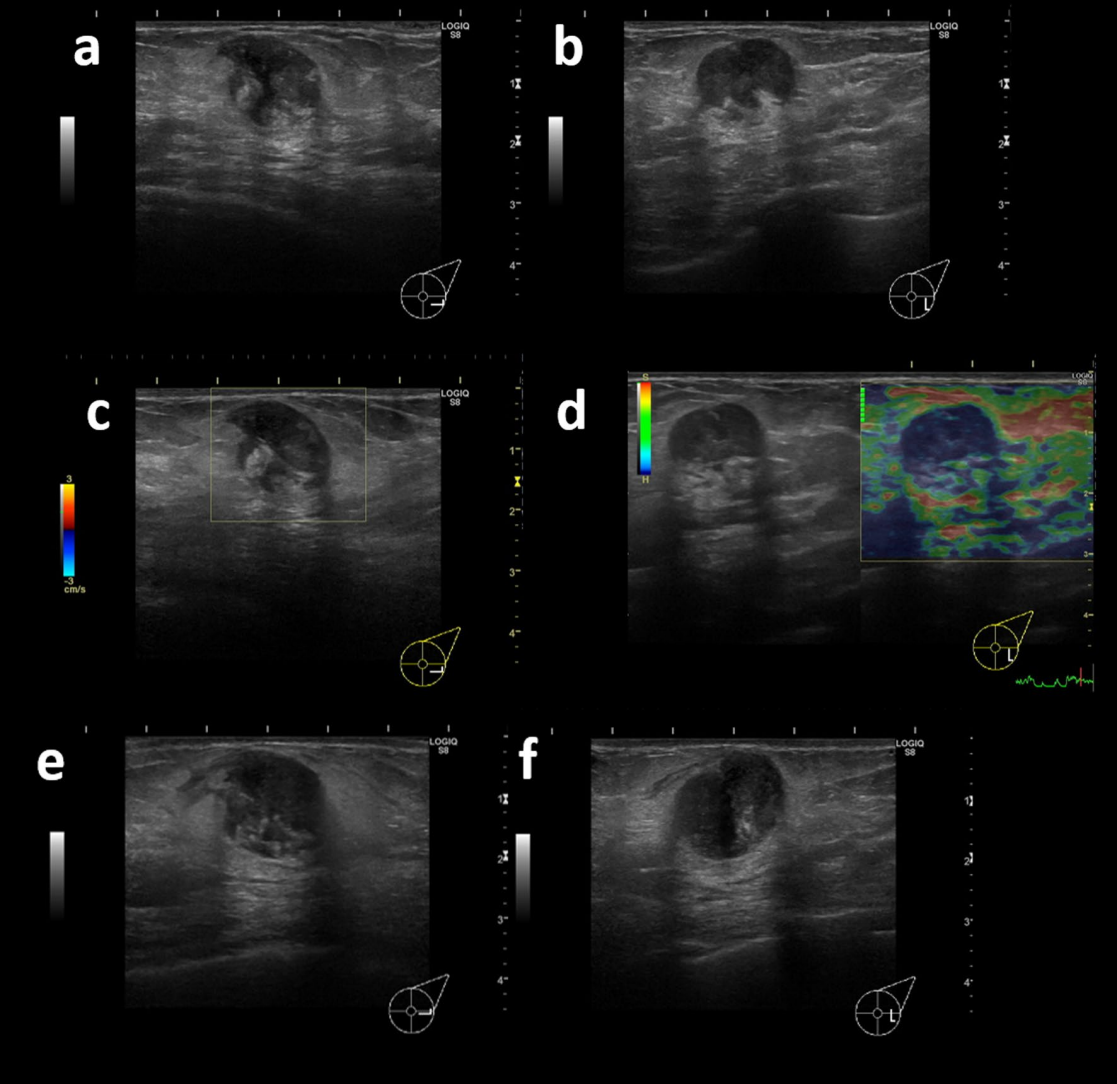
Breast ultrasonography. **a** US reveals a solid, round mass with circumscribed margins in the lower outer quadrant of the left breast at 4 o'clock, measuring 16 × 16 × 15 mm. The internal echo of the tumor was a mixture of relatively uniform hypoechoic areas with posterior enhancement, and heterogeneous hyperechoic areas. Color Doppler showed no blood flow in the tumor. **b** At 2 months follow-up ultrasonography, revealed an increase in the size of the mass to 27 × 26 × 19-mm

A 95-year-old woman visited a local clinic as soon as she became aware of a mass in her left breast. After undergoing mammography and breast ultrasonography, the patient was referred to our department for suspected breast cancer. On clinical examination, the mass measured 2 cm in diameter and was well defined and mobile, with the normal overlying skin and nipple-areola. The axillary lymph nodes were not palpable. No café-au-lait spots and no neurofibromas on the skin were observed. The patient had no family history of breast cancer or any other comorbidities. Mammography revealed a well-defined mass in the mid-lateral area of the left breast (Fig. 1). The shape of the mass is round, hyperdense to the breast glandular tissue, it was not accompanied by calcification. No abnormal skin thickening, nipple retraction or significant axillary lymphadenopathy is noted. Breast ultrasonography revealed a 16 × 16 × 15-mm round mass with posterior enhancement in the lower outer quadrant of the left breast at 4 o’clock, approximately 5.0 cm from the nipple, corresponding to the mammographic mass (Fig. 2a, b). The internal echo of the tumor was a mixture of relatively uniform hypoechoic areas with posterior enhancement and heterogeneous hyperechoic areas. Color Doppler revealed no blood flow signal in the tumor (Fig. 2c). Elastography was almost uniformly blue, indicating a poorly deformable lesion (Fig. 2d). Tsukuba Elasticity Score was 4. The tumor markers such as carcinoembryonic antigen and carbohydrate antigen 15-3, were within normal limits. She underwent a core needle biopsy. The pathological findings revealed a spindle cell lesion with no malignant findings. At 2 months follow-up, repeat breast ultrasonography revealed that the mass had increased in the size (27 × 26 × 19 mm) (Fig. 2e, f). A repeat core needle biopsy, however, provided no particularly new information. The tumor was resected without exposure, due to the tumor’s tendency to grow, and the malignant nature of the tumor could not be completely ruled out.

**Fig. 3:**
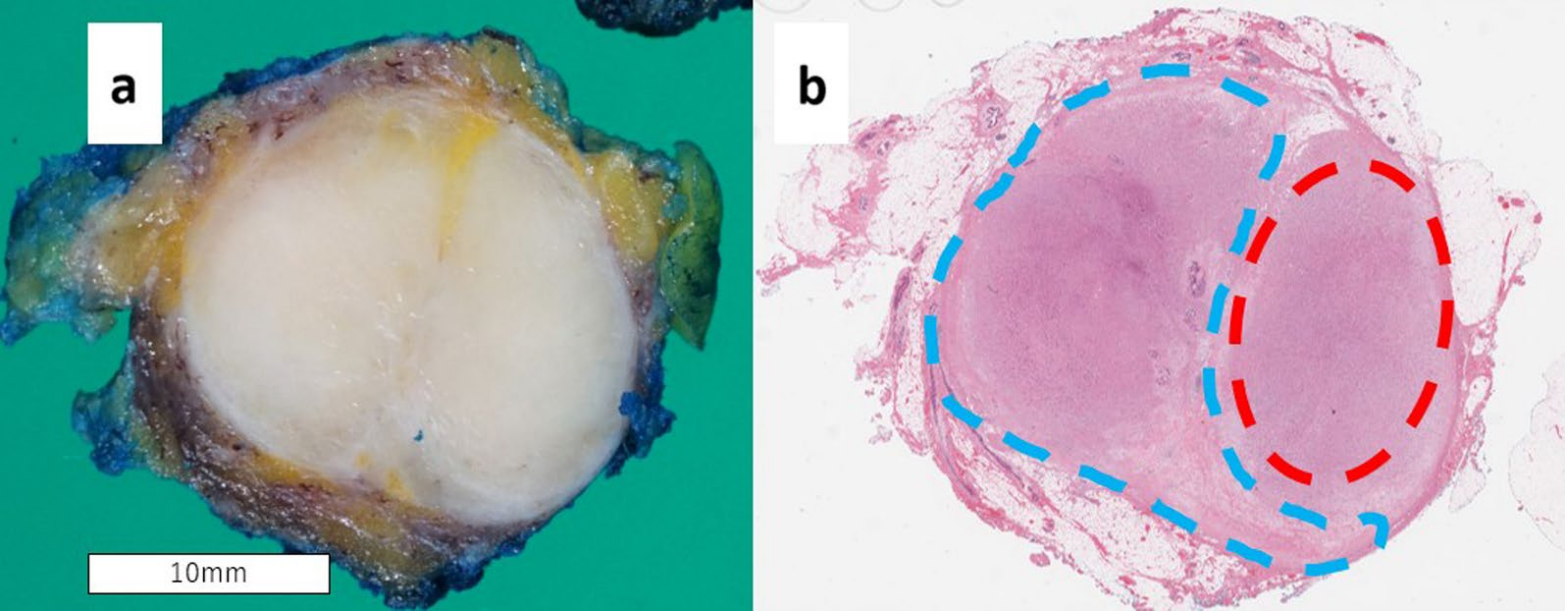
**a** Macroscopic appearance of the tumor. **b** Panoramic view of the tumor (hematoxylin and eosin staining). Spindle cells are densely packed in the area marked by the red dotted line. The blue dotted area is sparsely mixed with non-spindle cells, that is adenosis

**Fig. 4:**
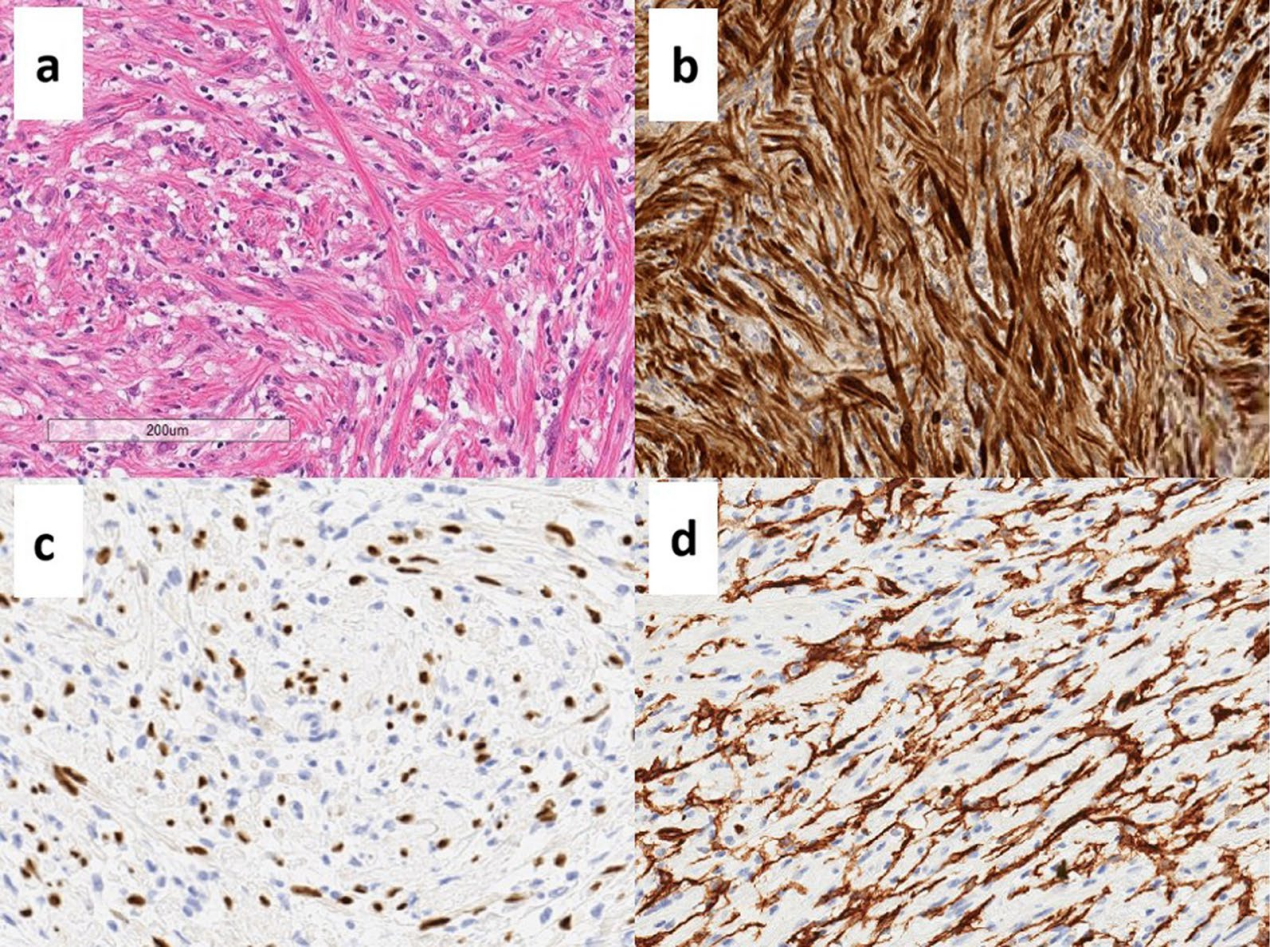
Immunohistological findings of the resected neurofibroma. **a** The majority of tumors show proliferation of fibroblast-like spindle cells with shredded-carrot collagen bundles (hematoxylin and eosin staining). **b** S100 was positive for spindle cells. **c** SOX10 was partially positive in spindle cells. **d** CD34 positivity in the spindle cells

**Fig. 5:**
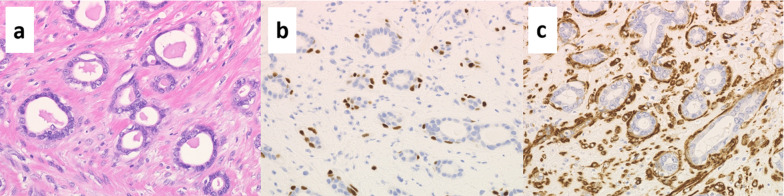
Immunohistological findings of the blue dot area. **a** The bilayer nature of luminal cells and myoepithelial cells were observed. **b** p63 was negative. **c** αSMA was positive in myoepithelial cells

Macroscopically, the tumor measured 20 × 19 × 17 mm and had a white cut surface (Fig. 3a). The results of the hematoxylin and eosin staining are shown in Fig. 3b. The area indicated by the red dotted line was densely populated with spindle-shaped cells and shredded-carrot collagen bundles (Fig. 4a), and this area was homogeneous with posterior enhancement on ultrasound. Immunohistochemical antibody markers (S100, SOX10, and cluster of differentiation [CD] 34) were positive in the spindle cells (Fig. 4b–d), indicating a neurofibroma. In contrast, the blue dotted area (Fig. 3b) maintained the bilayer nature of luminal cells and myoepithelial cells, which led to the diagnosis of adenosis. Hence, a histological diagnosis of neurofibroma with adenosis was made. At 6 months follow-up, no recurrent lesions were found (Fig. 5).

## Discussion

Neurofibromas arising within the breast parenchyma are extremely rare, and only three cases have been reported in which the neurofibroma was clearly identifiable on imaging as a neurofibroma within the mammary tissue [6–8]. Of these three case reports, two had tumors locating closer to the chest wall than within the breast [6, 7]. This is one of the few reports in which the rare intramammary tumor could clearly be identified within the breast gland by imaging studies. No difference between males and females in the incidence of this disease is observed. The age of affected patients is reported to range widely from 4 to 77 years [9, 10], and our patient is the oldest. Mammography of this condition shows a round or oval well-defined mass. Ultrasound examination reveals a well-defined round lesion, which can be either hypoechoic or anechoic with posterior acoustic enhancement [6]. Regarding the findings with Color Doppler, half of the patients were blood flow-rich, and half were hypovascular. However, in our patient, ultrasound images showed internal heterogeneity in one part of the mass. This was because the mass also contained adenosis. We searched PubMed for the words “neurofibroma” “breast” “adenosis” and found no precedent. The reason for adenosis accompanying the neurofibroma is unknown. In addition, the association between the presence of adenosis inside the tumor and its growth in a short period of time is unknown.

In case reports of breast neurofibroma, tumor resection was preferred and the patient was treated with tumor resection. In general, solitary neurofibromas are associated with a low local recurrence rate if completely excised [12]. There are few reports of long-term follow-up of breast neurofibromas, and the risk of malignant transformation is unknown. In contrast, the risk of malignant transformation of a neurofibroma in patients with NF1 is approximately 4.6% [11]. Further case accumulation of breast neurofibroma in non-neurofibromatosis is necessary. A case has been reported in which the tumor gradually grew to 2200 g in size after two years of follow-up [13]. The only reference describing the rate of enlargement was this single case report, which did not describe the size of the tumor at the time of initial diagnosis. In the present case, the volume increased 3.5-fold in approximately 2 months after the patient became aware of the breast mass. Most benign tumors such as fibroadenoma do not change in the short time. In this case, needle biopsy is mainly spindle cell proliferation, not typical fibroadenoma, which is the reason for the short follow up. If a benign tumor with spindle cell lesion is found to be enlarged, we recommend tumor resection.

In addition, diagnosis breast needle biopsy is often difficult because of the small sample amount and wide range of differential diagnoses. The differential diagnosis of a neurofibroma includes neuroma, schwannoma, and bland non-nerve sheath spindle cell lesions [14]. Microscopically, a typical neurofibroma consists of bland spindle cells and fibroblasts dispersed in loose collagen fibers that condense to form a shredded carrot-like appearance. Neurofibromas lack nuclear palisading and Verocay bodies, alternating Antoni A and Antoni B areas, and prominent hyalinized vessels. By immunohistochemistry, a proportion of spindled cells express S100 and SOX10, CD34 stains a subset of stromal cells, but cytokeratins, ER, actin, and desmin are negative.

## Conclusions

Ultrasound and pathological images revealed an extremely rare case of neurofibroma combined with adenosis. Tumor resection was performed because the tumor was growing, making definitive diagnosis with needle biopsy difficult. Immunohistochemistry was used to confirm the diagnosis.

## Data Availability

All data generated or analyzed during this study are included in this published article [and its additional information files].

## Abbreviations

CK: Cytokeratin
ER: Estrogen receptor
CD: Cluster of differentiation
